# Digital Quantum Simulation of Wavepacket Correlations in a Chemical Reaction

**DOI:** 10.3390/e28020144

**Published:** 2026-01-28

**Authors:** Shah Ishmam Mohtashim, Sabre Kais

**Affiliations:** 1 Department of Chemistry, North Carolina State University, Raleigh, NC 27695, USA; smohtas@ncsu.edu; 2 Department of Electrical & Computer Engineering, North Carolina State University, Raleigh, NC 27695, USA

**Keywords:** correlation functions, digital quantum simulation, wavepacket propagation, quantum algorithms

## Abstract

We present hybrid quantum–classical algorithms to compute time-dependent Møller wavepacket correlation functions via digital quantum simulation. Reactant and product channel wavepackets are encoded as qubit states, evolved under a discretized molecular Hamiltonian, and their correlation is reconstructed using both a modified Hadamard test and a multi-fidelity estimation (MFE) protocol. The method is applied to the collinear H + H_2_ exchange reaction on a London–Eyring–Polanyi–Sato potential energy surface. Quantum-estimated correlation functions show quantitative agreement with high-resolution classical wavepacket simulations across the full time domain, reproducing both short-time scattering peaks and long-time oscillatory dynamics. The ancilla-free MFE protocol achieves matching results with reduced circuit depth. These results provide a proof of principle that digital quantum circuits can be used to accurately calculate the wavepacket correlation functions for a benchmark chemical reaction system.

## 1. Introduction

Understanding how quantum states evolve in time lies at the foundation of modern chemical physics, condensed matter theory, and quantum information science. Many dynamical processes of physical interest—including energy transfer and wavepacket propagation—can be characterized through time-dependent correlation functions, which quantify the overlap between quantum states as they evolve under a given Hamiltonian. These correlation functions provide direct insights into coherence, interference, and the temporal structure of molecular motion, making them essential tools for analyzing quantum dynamical systems.

Classically computing such correlations often requires propagating high-dimensional wavefunctions on strongly coupled potential energy surfaces, a task that becomes increasingly demanding as the system size grows [1]. Quantum computers offer a natural platform for simulating unitary dynamics, motivating the development of digital quantum algorithms capable of estimating dynamical overlaps with scalability beyond classical limits [2,3,4]. Recent progress in quantum hardware and circuit design has made it possible to explore such algorithms in realistic molecular contexts, even within the constraints of near-term devices [5,6,7,8,9].

In this work, we present a hybrid quantum–classical framework for computing time-dependent correlation functions using digital quantum simulation. Our method implements the time-dependent Møller operator construction of reactant and product wavepackets on quantum circuits, allowing the correlation between two wavepackets to be expressed as a measurable quantum expectation value. We employ two complementary correlation estimation strategies: the modified Hadamard test, which uses an ancilla qubit to extract complex-valued overlaps, and the multi-fidelity estimation (MFE) protocol, which performs overlap estimation without controlled operations [10]. These approaches provide a flexible set of tools with reasonable circuit depth while retaining full access to the real and imaginary components of the correlation function.

To demonstrate the method, we consider the collinear H + H_2_ reaction system, modeled through wavepacket propagation on a London–Eyring–Polanyi–Sato (LEPS) potential energy surface. Vibrational motion is represented using Morse oscillator eigenfunctions, while translational motion is encoded in Gaussian wavepackets. The full molecular Hamiltonian is discretized on a two-dimensional grid in bond coordinates and mapped to qubits. Time evolution is implemented through unitary gates, and the resulting quantum-estimated correlation functions are benchmarked against high-resolution classical simulations of standard quantum calculations.

The results show close agreement between the quantum circuit and classical simulations of standard quantum calculations of correlation functions across the full time domain, capturing the expected long-time behavior and phase evolution of the wavepackets. This demonstrates that digital quantum simulation can reliably reproduce dynamical features encoded in time-dependent overlaps [11].

Overall, the present study establishes correlation function computation as a practical and informative intermediate step toward the quantum simulation of molecular dynamics. The combination of ancilla-based and ancilla-free estimation schemes provides circuit flexibility compatible with near-term quantum hardware. These results highlight the potential of digital quantum simulation to serve as a scalable framework for studying quantum dynamics and lay the groundwork for future extensions to higher-dimensional systems and more complex molecular processes.

Previous quantum simulation studies have addressed molecular ground-state energies, vibrational spectra, and short-time dynamics; however, the direct computation of time-dependent correlation functions using Møller operators has remained largely unexplored within a fully digital quantum framework [10,12]. The present work fills this gap by introducing a circuit-level formulation of channel-resolved wavepacket correlations and benchmarking two complementary quantum circuit estimators against classical computer simulations of standard quantum mechanical correlation calculations.

From an information-theoretic viewpoint, the Møller correlation $C_{\nu_{p},\nu_{r}}\left(t\right)$ is a Loschmidt echo-type overlap that quantifies how much dynamical information about the reaction is retained in the evolving wavepacket. Its magnitude |C(t)| is equivalent to the quantum fidelity between reactant and product preparations [13].

To summarize, in this work, we (i) formulate time-dependent Møller wavepacket correlation functions as quantum-measurable overlaps; (ii) conceptually experiment with a modified Hadamard test and a multi-fidelity estimation (MFE) protocol as prototype implementations, primarily to confirm the consistency between the measured data and the predicted theoretical bounds for the H + H_2_ reaction; and (iii) perform conceptual data confirmation by comparing the resulting channel-resolved correlations with high-resolution standard quantum calculations on classical computer wavepacket propagation and correlation. Our aim is proof of principle: we show that chemically meaningful overlaps can be computed on digital quantum circuits and are compatible with near-term hardware constraints.

## 2. Model: Collinear Hydrogen Exchange Reaction

$H_{a}+H_{b}H_{c}\;⟶\;H_{a}H_{b}+H_{c}$ serves as a fundamental prototype for studying reactive molecular dynamics [14,15,16,17,18]. Owing to its minimal number of degrees of freedom and well-established potential energy surfaces, the model provides an ideal setting to test new numerical and quantum simulation frameworks. Despite its simplicity, the system exhibits key dynamical features found in more complex reactions, including barrier crossing, transient energy redistribution, and vibrational state selectivity.

### 2.1. Bond and Jacobi Coordinates

**Figure 1:**
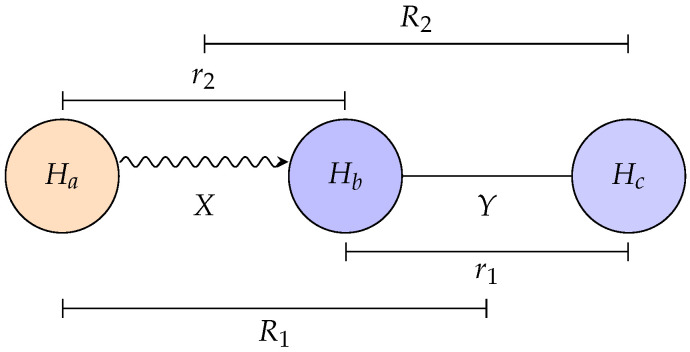
Collinear geometry of the H_a_ + H_b_H_c_→ H_a_H_b_ + H_c_ reaction, showing the bond coordinates (X, Y) and corresponding Jacobi coordinates $\left(R_{1},\;r_{1}\right)$ and $\left(R_{2},\;r_{2}\right)$ used to describe the reactant and product arrangements.

In a collinear triatomic system, shown in Figure 1, two coordinate systems are commonly used to represent nuclear motion.

The first is Jacobi coordinates, which naturally describe individual channels. For the H + H_2_ system, the reactant arrangement channel is described by $\left(R_{1},\;r_{1}\right)$, where $r_{1}$ is the bond length of the diatomic H_2_, and $R_{1}$ is the separation between the incoming H atom and the diatomic center of mass. Similarly, the product arrangement channel is described by $\left(R_{2},\;r_{2}\right)$, where $r_{2}$ is the bond length of the newly formed H_2_, and $R_{2}$ is the distance from the scattered H atom to the product diatomic center of mass.

Channel definition: In this work, channel refers to a choice of vibrational quantum numbers associated with the asymptotic reactant and product arrangements. We label the reactant vibrational quantum number $\nu_{r}$ and the product vibrational quantum number $\nu_{p}$. Throughout this study, the initial reactant is prepared in its vibrational ground state $\nu_{r}=0$, and we consider two product channels corresponding to $\nu_{p}=0$ (vibrationally elastic scattering) and $\nu_{p}=1$ (transition to the first vibrationally excited product state).

The second is Bond coordinates, defined simply as the interatomic distances

(1) $$X=r_{12},\;Y=r_{23}$$

This representation provides a single (X, Y) grid on which both reactant and product wavepackets can be expressed simultaneously.

The two descriptions are related by a straightforward linear transformation. Specifically, the bond coordinates (X, Y) can be obtained from either the reactant Jacobi coordinates $\left(R_{1},\;r_{1}\right)$ or the product Jacobi coordinates $\left(R_{2},\;r_{2}\right)$ as

(2) $$\left(\begin{array}{c} X\\ Y \end{array}\right)=\left(\begin{array}{c} R_{1}-\frac{1}{2}r_{1}\\ r_{1} \end{array}\right)=\left(\begin{array}{c} r_{2}\\ R_{2}-\frac{1}{2}r_{2} \end{array}\right)$$

In practice, the initial wavepackets are prepared in Jacobi coordinates, reflecting their channel character, and then transformed into the common bond coordinate grid (X, Y). All subsequent propagation and correlation function calculations are carried out in this bond coordinate representation, which unifies the treatment of reactant and product channels.

### 2.2. Structure of the Wavepacket

For each channel, the wavepacket is constructed as a product of three factors:

(3) $$\Psi \left(R,\;r\right)=N_{G}\;\psi_{\mathrm{vib}}\left(r\right)\;\exp\;\left[-\Delta k^{2}{\left(R-R_{0}\right)}^{2}\right]\;\exp\;\left[ik_{0}\left(R-R_{0}\right)\right]$$

Here,

1. $\psi_{\mathrm{vib}}\left(r\right)$ describes the internal vibration of the diatomic molecule (H_2_);
2. The Gaussian envelope $\exp[-\Delta k^{2}{\left(R-R_{0}\right)}^{2}]$ ensures the localization of the wavepacket in the translational coordinate *R*, centered at $R_{0}$;
3. The carrier plane wave $\exp[ik_{0}\left(R-R_{0}\right)]$ gives the packet a mean momentum $k_{0}$, directed either inward (reactant) or outward (product) relative to the interaction region.

The prefactor is chosen to guarantee square-integrability and to match the conventional normalization of Gaussian wavepackets:

(4) $$N_{G}={\left(\frac{2\Delta k^{2}}{\pi }\right)}^{1/4}.$$

#### Vibrational Component: Morse Oscillator

The vibrational eigenstates of H_2_ are modeled using the Morse oscillator, which provides a realistic one-dimensional description of bond stretching. The eigenfunction for vibrational quantum number *n* is

(5) $$\psi_{\mathrm{vib}}\left(r\right)=N_{n}\;z^{\lambda -n-\frac{1}{2}}\;e^{-z/2}\;L_{n}^{\left(2\lambda -2n-1\right)}\left(z\right),$$

where $L_{n}^{\left(\alpha \right)}\left(z\right)$ are generalized Laguerre polynomials. The dimensionless coordinate *z* and parameter λ are defined as

(6) $$z=2\lambda e^{-a(r-r_{e})},\;\lambda =\frac{\sqrt{2\mu D_{e}}}{a\hbar }.$$

Here,

- $D_{e}$ is the dissociation energy of the bond;
- *a* is an inverse range parameter controlling the curvature of the potential at equilibrium;
- $r_{e}$ is the equilibrium bond length;
- μ is the reduced mass of the diatomic fragment (for H-H, $\mu \approx m_{H}/2\approx 918\;m_{e}$ in atomic units).

The normalization constant $N_{n}$ is fixed by the orthonormality of Morse eigenstates:

(7) $$N_{n}=\sqrt{\frac{n!\;(2\lambda -2n-1)\;a}{\Gamma (2\lambda -n)}}.$$

### 2.3. Interaction Potential

To model the nuclear motion in the collinear H + H_2_ reaction, we employ a London–Eyring–Polanyi–Sato (LEPS)-type potential energy surface. In this construction, each pairwise H-H interaction is described by an effective diatomic potential, and the three pair potentials are then combined into a three-center form that interpolates smoothly between reactant and product valleys while generating a single barrier along the exchange path. The LEPS ansatz captures the essential barrier–well topology of the H_3_ system with relatively few parameters and has long served as a benchmark surface for time-dependent studies of hydrogen exchange. In the following, we summarize the two-body building blocks and their London-type three-body combination, which are used to define the interaction potential in our simulations.

#### 2.3.1. Two-Body Forms: Morse and Anti-Morse Potentials

Each pairwise interaction is represented by two effective potential forms:

(8) $$\sigma_{1}(r)=D_{e}{\left(1-e^{-\beta (r-r_{e})}\right)}^{2},$$

(9) $$\sigma_{3}\left(r\right)=\frac{1}{2}D_{e}\;\left(e^{-2\beta (r-r_{e})}+2\;e^{-\beta (r-r_{e})}\right),$$

where $\sigma_{1}$ is the standard Morse potential describing the covalent bond well, and $\sigma_{3}$ is an anti-Morse counterpart that emphasizes the short-range repulsive character while retaining exponential decay. Following the London construction, these are combined into symmetric and antisymmetric forms, interpreted as approximate Coulomb and exchange contributions:

(10) $$Q(r)=\frac{1}{2}\left(\sigma_{1}\left(r\right)+\sigma_{3}\left(r\right)\right),$$

(11) $$\alpha (r)=\frac{1}{2}\left(\sigma_{1}\left(r\right)-\sigma_{3}\left(r\right)\right).$$

#### 2.3.2. Three-Center London Combination

For the collinear triatomic system, we evaluate *Q* and α on all three bonds. The London-type three-body combination is

(12) $$V_{L}\left(r_{12},\;r_{23},\;r_{13}\right)=Q_{a}+Q_{b}+Q_{c}+s\;\frac{1}{\sqrt{2}}\;\sqrt{{\left(\alpha_{a}-\alpha_{b}\right)}^{2}+{\left(\alpha_{a}-\alpha_{c}\right)}^{2}+{\left(\alpha_{b}-\alpha_{c}\right)}^{2}},$$

where s=±1 sets the sign of the exchange-like term. In this work, we take s=−1, which lowers the energy when one bond is near its covalent minimum while the others are elongated, producing a barrier–well profile consistent with the H + H_2_ exchange reaction.

The resulting surface exhibits a single saddle corresponding to the collinear exchange barrier, with reactant and product valleys along the asymptotic channels. The qualitative topology of $V_{L}$ agrees with benchmark LEPS representations used in classical and semiclassical scattering studies [19,20,21]. Historically, this construction corresponds to the London–Eyring–Polanyi–Sato (LEPS) potential energy surface, which was evaluated empirically for H + H_2_ by Cashion and Herschbach [22] and remains a benchmark model for testing reaction models [14,15].

For our chemical reaction model, we use (in a.u.)

(13) $$D_{e}\approx 0.174,\;r_{e}\approx 1.41,\;\beta \approx 1.028,$$

which reproduce a qualitatively reasonable covalent well for each pair in Equation (8) and generate a barrier profile consistent with collinear exchange under Equation (12).

**Figure 2:**
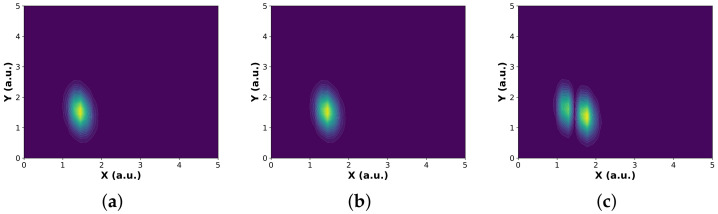
Spatial structure of the reactant and product wavepackets on the common bond coordinate grid (X, Y). Here, *X* and *Y* denote the two H-H bond lengths (in atomic units). (**a**) Incoming reactant Møller wavepacket $\Psi_{v=0}^{+}\left(X,\;Y\right)$. (**b**) Outgoing product Møller wavepacket $\Psi_{v^{\prime }=0}^{-}\left(X,\;Y\right)$ for the vibrational ground state. (**c**) Outgoing product Møller wavepacket $\Psi_{v^{\prime }=1}^{-}\left(X,\;Y\right)$ for the first vibrationally excited state. The reactant wavepacket is initialized with central momentum $k_{0}=-8.2$ a.u. and momentum width Δk=1.25 a.u., while the product wavepackets have $k_{0}=+8.2$ a.u. with the same Δk. The initial wavepacket center is located at $R_{0}=2.25$ a.u. in both channels.

**Figure 3:**
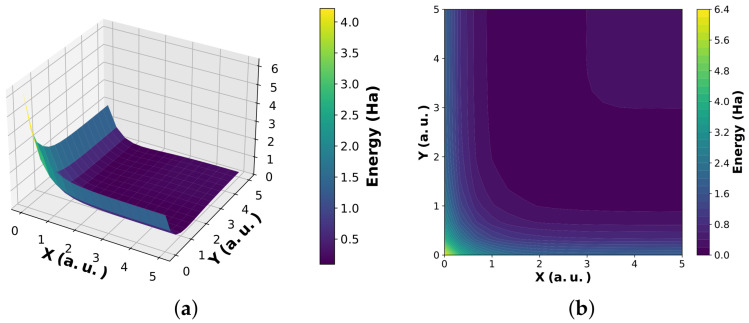
London–Eyring–Polanyi–Sato (LEPS) potential energy surface for the collinear H_a_ + H_b_H_c_ system in bond coordinates (X, Y). Here, *X* and *Y* denote the H-H bond lengths (in atomic units). (**a**) Three-dimensional representation of the LEPS potential V(X, Y). (**b**) Contour plot of the same surface. Energies are shown in Hartree atomic units.

The spatial structure of the reactant and product wavepackets in bond coordinates (X, Y) is shown in Figure 2, and the corresponding LEPS potential energy surface governing the interaction dynamics is shown in Figure 3.

### 2.4. Discrete Kinetic Energy Operator in Bond Coordinates

The total Hamiltonian on the (X,Y) bond coordinate grid requires, in addition to the London potential, a representation of the nuclear kinetic energy operator. For a collinear triatomic system with equal masses (hydrogen atoms), the kinetic part of the Hamiltonian in bond coordinates can be expressed as

(14) $$\hat{T}=\frac{1}{m_{H}}\left(-\frac{\partial^{2}}{\partial X^{2}}-\frac{\partial^{2}}{\partial Y^{2}}+\frac{\partial^{2}}{\partial X\;\partial Y}\right),$$

where $m_{H}$ is the mass of a hydrogen atom in atomic units. The first two terms correspond to the standard Laplacian in *X* and *Y*, while the mixed derivative $\partial_{XY}$ arises due to the non-orthogonal nature of the bond coordinate system.

## 3. Methods: Møller Operator Formulation of Wavepacket Propagation

In the time-dependent formulation of wave propagation theory, the central objects are the Møller operators, which connect asymptotic free states to interacting scattering states. Let $H_{0}$ denote the channel Hamiltonian that governs the non-interacting asymptotic dynamics, and let $H=H_{0}+V$ be the full Hamiltonian including the interaction potential *V*. The Møller operators are defined as

(15) $$\Omega^{\pm }=\lim_{t\to \mp \infty }e^{iHt}e^{-iH_{0}t}.$$

Physically, $\Omega^{+}$ maps a free state prepared in the distant past to the corresponding incoming reactant state, while $\Omega^{-}$ maps a free state prepared in the distant future to the corresponding outgoing product state. Thus, the ‘+’ sign encodes incoming boundary conditions and the ‘−’ sign encodes outgoing boundary conditions.

Acting with $\Omega^{\pm }$ on an asymptotic channel basis state $|k_{\gamma },\gamma \rangle$, characterized by a relative momentum $k_{\gamma }$ and an internal state label γ, produces the interacting scattering state

(16) $$|\Psi_{\gamma }^{\pm }\rangle =\Omega^{\pm }|k_{\gamma },\gamma \rangle .$$

These states retain the asymptotic character of the channel γ far from the interaction region but evolve according to the full Hamiltonian *H* near the reaction zone.

In the present work, the term channel refers to a choice of vibrational quantum numbers for the reactant and product H-H bond. We label the reactant vibrational level $\nu_{r}$ and the product vibrational level $\nu_{p}$. Throughout, we consider an initial reactant prepared in its vibrational ground state, $\nu_{r}=0$, and we focus on two product channels: $\nu_{p}=0$ (vibrationally elastic scattering) and $\nu_{p}=1$ (transition to the first vibrationally excited product state) [23]. The corresponding channel-resolved time-dependent correlation function is

(17) $$C_{\nu_{p},\nu_{r}}\left(t\right)=\langle \Psi_{\nu_{p}}^{-}\left|e^{-iHt}\right|\Psi_{\nu_{r}}^{+}\rangle .$$

For the choices above, $|C_{0,0}\left(t\right)|$ measures the time-dependent probability amplitude for the system to remain in the vibrational ground state, while $|C_{1,0}\left(t\right)|$ measures the amplitude for an inelastic transition into the first excited vibrational channel. The peaks and long-time oscillations of these quantities, shown later in the Section 6, encode the dynamical signatures of barrier crossing and vibrational recurrences in the H + H_2_ exchange reaction [16,24,25].

In other words, C(t) is the overlap between a reactant-like wavepacket $\Omega^{+}|\phi \rangle$ propagated forward in time by *t* and a product-like wavepacket $\Omega^{-}|\phi \rangle$ propagated backward in time by *t*, with both wavepackets evaluated at a common reference time. Varying *t* traces the dynamical progression of the scattering process, with the transient reaction event reflected in the behavior of C(t) near t≈0. This correlation function contains the same physical information as the traditional *S*-matrix elements, but it is naturally expressed in terms of time evolution operators and overlaps, which are directly implementable on a quantum computer as unitary circuits. It is precisely this quantity that we encode and measure with the quantum algorithms described in the following sections.

## 4. Standard Quantum Calculations

To obtain the results reported here, we used a uniform bond coordinate grid with $N_{X}=N_{Y}=16$ points spanning 0≤X,Y≤5a.u., giving a Hilbert-space dimension $D=N_{X}N_{Y}=256$. The time-dependent correlations were evaluated on a uniform time grid t∈[−1000, 4000]a.u. with step size Δt=50a.u., which resolves both the short-time scattering peak and long-time vibrational recurrences [15,25,26,27,28].

**Figure 4:**
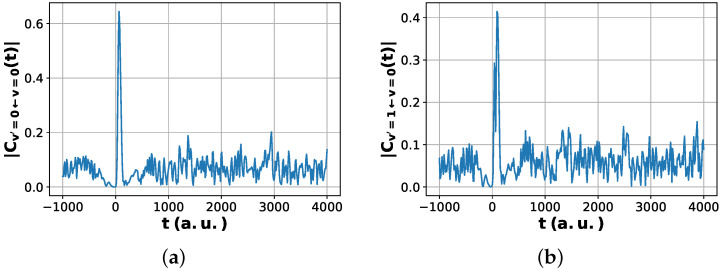
Time-dependent magnitude of the channel-resolved correlation functions with the reactant prepared in its vibrational ground state. (**a**) $|C_{0,0}\left(t\right)|$ corresponding to transitions from the reactant vibrational ground state to the product vibrational ground state. (**b**) $|C_{1,0}\left(t\right)|$ corresponding to transitions from the reactant vibrational ground state to the first vibrationally excited product channel. The sharp peak near t≈0 reflects the transient overlap during the scattering event, followed by long-time oscillatory fluctuations associated with outgoing wavepacket dynamics.

These standard quantum calculations on classical computer simulations, shown in Figure 4, provide a reference for assessing the quantum algorithms. In particular, they show that physically relevant information about the reaction and product overlap and crossing, partial transmission into the product channel, and long-time vibrational recurrences is encoded in the time-resolved overlaps $C_{\nu_{p},\nu_{r}}\left(t\right)$. The key question that we address in the following sections is whether digital quantum circuits can reproduce these features with sufficient accuracy to serve as reliable surrogates for standard quantum calculations on classical computer wavepacket propagation.

## 5. Quantum Algorithms and Implementation

### 5.1. Hamiltonian Construction in the Grid Basis

The total Hamiltonian for the collinear H + H_2_ reaction is written as

(18) $$H=T_{X}+T_{Y}+T_{XY}+V\left(X,\;Y\right),$$

where *X* and *Y* denote the bond coordinates, $T_{X}$ and $T_{Y}$ are the kinetic energy operators, and V(X, Y) is the interaction potential. The asymptotic Hamiltonian is given by

(19) $$H_{0}=T_{X}+T_{Y}+T_{XY},$$

corresponding to free propagation in the absence of interaction. The kinetic energy operator in bond coordinates contains both diagonal and mixed derivative terms,

(20) $$T=T_{X}+T_{Y}+T_{XY},$$

where $T_{X}$ and $T_{Y}$ denote the second derivatives with respect to *X* and *Y*, respectively, and $T_{XY}$ is the mixed kinetic term arising from the coordinate transformation from Jacobi to bond coordinates. This mixed term encodes the kinematic coupling between the two bond lengths and is essential in accurately describing the collinear three-body dynamics.

Both Hamiltonians are represented on a uniform two-dimensional grid in (X, Y). The kinetic energy operators are discretized using second-order finite difference approximations, while the interaction Hamiltonian is obtained by evaluating the potential energy surface V(X, Y) pointwise on the grid. This representation captures both the translational motion along the reaction coordinate and the vibrational dynamics of the diatomic fragment.

The resulting Hamiltonian matrices are then mapped to qubit operators by expressing them as linear combinations of Pauli strings, which are subsequently used to construct the time evolution operator $U\left(t\right)=e^{-iHt}$ in the quantum circuit.

### 5.2. Grid-Based Representation of Channel Wavepackets

The reactant and product channel states, denoted by $\psi_{r}$ and $\psi_{p}$, incorporate both the translational motion along the reaction coordinate and the vibrational dynamics of the diatomic fragment. In our implementation, these states are represented using a first-quantized, grid-based encoding on a two-dimensional bond coordinate grid (X, Y), following the standard real-space discretization approach used in quantum simulations of molecular scattering [4].

Each channel wavepacket is initially defined in its natural Jacobi coordinates $\left(R_{\gamma },r_{\gamma }\right)$ as a separable product

(21) $$\psi_{\gamma }\left(R_{\gamma },r_{\gamma }\right)=\phi_{\nu_{\gamma }}\left(r_{\gamma }\right)\;\chi_{\gamma }\left(R_{\gamma }\right),$$

where γ=r,p labels the reactant and product channels, respectively. The vibrational component $\phi_{\nu_{\gamma }}\left(r_{\gamma }\right)$ is taken to be an eigenstate of the Morse potential, while the translational component $\chi_{\gamma }\left(R_{\gamma }\right)$ is chosen as a Gaussian wavepacket with central momentum $k_{0,\gamma }$ and momentum width Δk,

(22) $$\chi_{\gamma }\left(R_{\gamma }\right)={\left(\frac{2\Delta k^{2}}{\pi }\right)}^{1/4}\exp\;\left[-\Delta k^{2}{\left(R_{\gamma }-R_{0}\right)}^{2}\right]\exp\;\left[ik_{0,\gamma }\left(R_{\gamma }-R_{0}\right)\right].$$

To place both channels on a common computational grid, the Jacobi coordinates are expressed in terms of the bond coordinates (X, Y) appropriate for the collinear H + H_2_ geometry. For the reactant channel,

(23) $$r_{r}=Y,\;R_{r}=X+\frac{1}{2}Y,$$

while, for the product channel,

(24) $$r_{p}=X,\;R_{p}=Y+\frac{1}{2}X.$$

Using these channel-dependent coordinate maps, the continuous wavepackets are evaluated directly on the (X, Y) grid,

(25) $$\psi_{\gamma }\left(X,\;Y\right)=\psi_{\gamma }\;\left(R_{\gamma }\left(X,\;Y\right),\;r_{\gamma }\left(X,\;Y\right)\right).$$

The grid is discretized uniformly as $X_{i}\in \left[0,\;X_{\max}\right]$ and $Y_{j}\in \left[0,\;Y_{\max}\right]$, with spacings ΔX and ΔY. After discretization, each channel wavepacket is normalized according to

(26) $$\sum_{i,j}{\left|\psi_{\gamma }\left(X_{i},\;Y_{j}\right)\right|}^{2}\;\Delta X\;\Delta Y=1.$$

### 5.3. Qubit Encoding and State Preparation

For the grid size used here ($N_{X}=N_{Y}=16$), the system dimension D=256 maps to $n_{\mathrm{sys}}=8$ qubits. The modified Hadamard test therefore uses $n_{\mathrm{tot}}=n_{\mathrm{sys}}+1=9$ qubits (including the ancilla), whereas the MFE protocol uses $n_{\mathrm{tot}}=2n_{\mathrm{sys}}=16$ qubits without an ancilla.

Each bond coordinate is represented on a discrete grid of size $N_{x}\;\times \;N_{y}$ in the (X, Y) bond coordinate plane. The joint Hilbert space of these grid points is mapped to a register of $n_{\mathrm{qubits}}=\log_{2}\left(N_{x}N_{y}\right)$ qubits.

In this proof-of-principle work, we load these states using a dense state preparation unitary obtained from the classical amplitude vectors, i.e., we assume access to a circuit that maps |0…0〉 to $|\Psi^{\pm }\rangle$ exactly. The Gaussian envelope parameters, such as the central momentum $k_{0}$ and width Δk, are chosen so that the initial wavepacket is localized near a chosen bond length $R_{0}$ and carries sufficient kinetic energy to traverse the barrier on the LEPS potential.

This approach of loading states using a dense state preparation unitary is restricted to small Hilbert spaces and is used here exclusively for validation and benchmarking. We do not claim this method to be scalable; rather, it allows us to isolate and test the algorithmic structure of the correlation estimation protocols. Scalable implementations would instead employ structured or approximate state preparation techniques.

### 5.4. Modified Hadamard Test

To evaluate the time-dependent correlation function,

(27) $$C\left(t\right)=\langle \psi_{\mathrm{prod}}|e^{-i\hat{H_{0}}\tau_{0}}e^{+i\hat{H}\tau_{0}}e^{-i\hat{H}t}e^{-i\hat{H}\tau_{0}}e^{i{\hat{H}}_{0}\tau_{0}}|\psi_{\mathrm{react}}\rangle ,$$

we implement a modified Hadamard test circuit augmented with controlled state preparation unitaries for both the reactant and product wavepackets.

#### Circuit Structure

**Figure 5:**
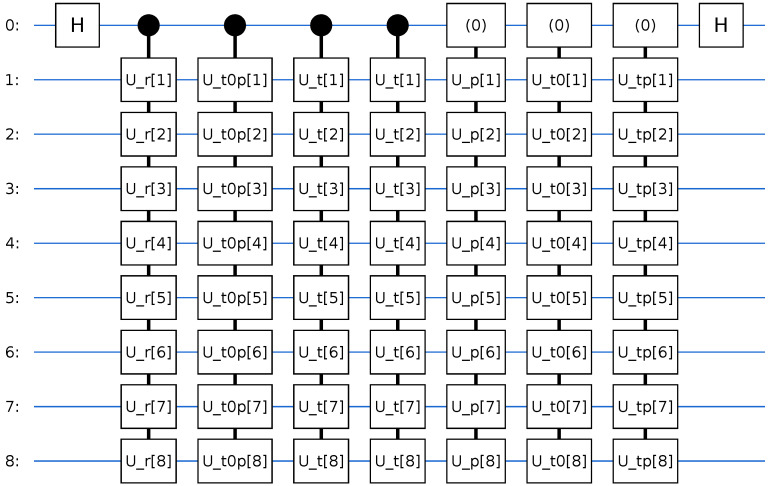
Quantum circuit used to measure the real part ReCvp,vr(t) of the channel-resolved correlation function via a modified Hadamard test. An ancilla qubit is first prepared in a superposition state and controls the preparation of the reactant and product wavepackets on the system register, together with the short-time Møller operator blocks and full time evolution unitaries. A final Hadamard on the ancilla, followed by measurement in the computational basis, yields the expectation value of $\sigma_{z}$ on the ancilla, which is proportional to ReCvp,vr(t).

Figure 5 shows the layout. The ancilla qubit is first prepared in a superposition state via a Hadamard gate. Conditioned on the ancilla, we then apply the following:

1. Controlled preparation of the reactant state $|\psi_{\mathrm{react}}\rangle$;
2. Controlled short-time Møller operator blocks $U_{H_{0}}\left(-\tau \right)$ and $U_{H_{0}}\left(+\tau \right)$ built from the non-interacting Hamiltonian ${\hat{H}}_{0}$;
3. A controlled real-time propagator $U_{H}\left(t\right)=e^{-i\hat{H}t}$;
4. Controlled preparation of the product state $|\psi_{\mathrm{prod}}\rangle$, implemented by flipping the ancilla into |1〉, applying the preparation;
5. Additional $U_{H}\left(\pm \tau \right)$ blocks acting only when the ancilla is in |0〉, ensuring the proper phase ordering in Equation (27).

Finally, another Hadamard gate on the ancilla and a projective measurement in the computational basis yield the expectation value

(28) ReC(t)=P(0)−P(1),

where P(0) and P(1) are the measured probabilities of finding the ancilla in states |0〉 and |1〉, respectively. This relation follows from the standard Hadamard test identity.

The quantum circuit used to measure the imaginary part ImCvp,vr(t) uses the same modified Hadamard test structure as in Figure 5, with an additional phase gate $S^{†}$ applied to the ancilla after the first Hadamard. This phase shift rotates the interference pattern on the ancilla such that the measured $\sigma_{z}$ expectation value is proportional to ImCvp,vr(t), while the underlying state preparation and time evolution blocks remain unchanged.

Each unitary block appearing in Figure 5 has a specific physical meaning. The operators $U_{r}$ and $U_{p}$ denote the state preparation unitaries that map the computational basis state |0…0〉 to the reactant and product channel wavepackets, $|\Psi_{r}\rangle$ and $|\Psi_{p}\rangle$, respectively. The unitary $U\left(t\right)=e^{-iHt}$ represents time evolution under the full interacting Hamiltonian, while $U_{H_{0}}\left(\pm \tau \right)=e^{\mp iH_{0}\tau }$ are short-time propagators generated by the asymptotic Hamiltonian and appear in the Møller operator construction. Controlled versions of these unitaries are used in the modified Hadamard test to encode the desired overlap into the ancilla qubit.

**Figure 6:**
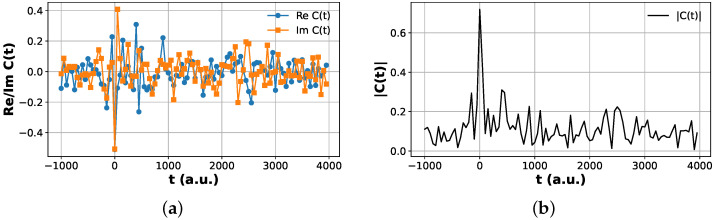
Hadamard test correlation function for the ground-state product channel. (**a**) Real and imaginary parts, ReCvp=0,vr=0(t) and ImCvp=0,vr=0(t), obtained from the modified Hadamard test protocol. (**b**) Magnitude $|C_{v_{p}=0,\;v_{r}=0}\left(t\right)|$ reconstructed from the same data. The short-time correlation peak and long-time oscillatory structure agree with the classical reference dynamics.

**Figure 7:**
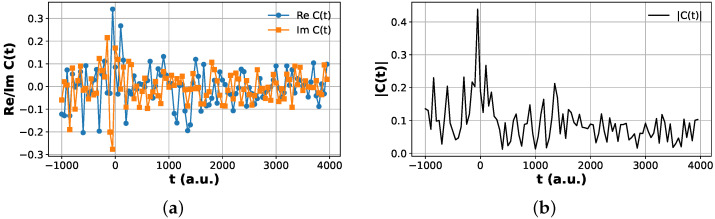
Hadamard test correlation function for the excited product channel $v_{p}=1$. (**a**) Real and imaginary parts, ReCvp=1,vr=0(t) and ImCvp=1,vr=0(t), obtained from the modified Hadamard test protocol. (**b**) Magnitude $|C_{v_{p}=1,\;v_{r}=0}\left(t\right)|$ reconstructed from the same data. The suppressed peak height relative to the $v_{p}=0$ channel reflects reduced excitation probability into the first vibrationally excited product state.

Plots of the Hadamard test correlation functions are shown in Figure 6 and Figure 7.

### 5.5. Multi-Fidelity Estimation (MFE) Method

The multi-fidelity estimation (MFE) method provides a resource-efficient way to estimate complex-valued quantum overlaps such as time-dependent correlation functions without relying on controlled unitaries or ancillary qubits. In this approach, instead of using the conventional Hadamard test, which demands extra circuit depth, the desired matrix element $\langle \phi_{i}|e^{-in\hat{H}\tau }|\phi_{j}\rangle$ is inferred from measurable state fidelities [29,30,31].

The method begins by preparing a normalized superposition state

(29) $$|\Psi_{\sup}\rangle =\frac{1}{\sqrt{2}}\left(\left|R\rangle +\right|\phi_{j}\rangle \right),$$

where the reference state |R〉 and the target state $|\phi_{j}\rangle$ belong to different symmetry sectors of the Hamiltonian. A second superposition,

(30) $$|\chi \rangle =\frac{1}{\sqrt{2}}\left(\left|R\rangle +\right|\phi_{i}\rangle \right),$$

is then constructed to serve as a projector state.

The overlap between these two states yields the fidelity

(31) $$F_{2}={\left|\langle \chi |\Psi_{\sup}\rangle \right|}^{2}=\frac{1}{4}{\left|\left(\langle \phi_{i}\left|+\langle R\right|\right)e^{-in\hat{H}\tau }\left(\left|R\rangle +\right|\phi_{j}\rangle \right)\right|}^{2},$$

while a direct fidelity measurement

(32) $$F_{1}={\left|\langle \phi_{i}|e^{-in\hat{H}\tau }|\phi_{j}\rangle \right|}^{2},$$

provides the magnitude $r=\sqrt{F_{1}}$ of the correlation function. The reference amplitude is independently evaluated as

(33) $$\langle R|e^{-i\hat{H}\tau }|R\rangle =r_{R}e^{i\theta_{R}},$$

where $r_{R}$ and $\theta_{R}$ denote the magnitude and phase of the reference state under time evolution. The phase of the target correlation function is then reconstructed via

(34) $$\theta =\cos^{-1}\;\left(\frac{4F_{2}-F_{1}-r_{R}^{2}}{2rr_{R}}\right)+\theta_{R},$$

yielding the complete complex overlap

(35) $$\langle \phi_{i}|e^{-in\hat{H}\tau }|\phi_{j}\rangle =re^{i\theta }.$$

Thus, the MFE protocol extracts both the amplitude and phase of time-dependent overlaps solely from fidelity measurements between evolved and reference states, avoiding controlled operations and making it especially suitable for near-term quantum hardware applications such as computing scattering matrix elements.

To summarize,

(36) $$\langle \phi_{i}\left|U\right|\phi_{j}\rangle =re^{i\theta }=\sqrt{F_{1}}e^{i[\cos^{-1}(\frac{4F_{2}-F_{1}-r_{R}^{2}}{2rr_{R}})+\theta_{R}]}$$

where $F_{1}=|\langle \phi_{i}\left|U\right|\phi_{j}{\rangle |}^{2}$ and $F_{2}={\left|\langle \chi |\Psi_{\sup}\rangle \right|}^{2}$ are measurable fidelities, while $r_{R}$ and $\theta_{R}$ denote the magnitude and phase of the reference state overlap $\langle R|U|R\rangle =r_{R}e^{i\theta_{R}}$. This formulation explicitly recovers both the amplitude and phase of the complex overlap from fidelity measurements alone, with the total measurement overhead scaling polynomially as $O(1/\varepsilon^{2})$ [10].

#### Circuit Structure

Advantages

- Avoids the use of controlled unitary operations and ancilla qubits;
- Reduces the circuit depth, enabling execution on Noisy Intermediate-Scale Quantum (NISQ) devices;
- Allows the efficient and accurate estimation of time correlation functions and scattering amplitudes.

**Figure 8:**
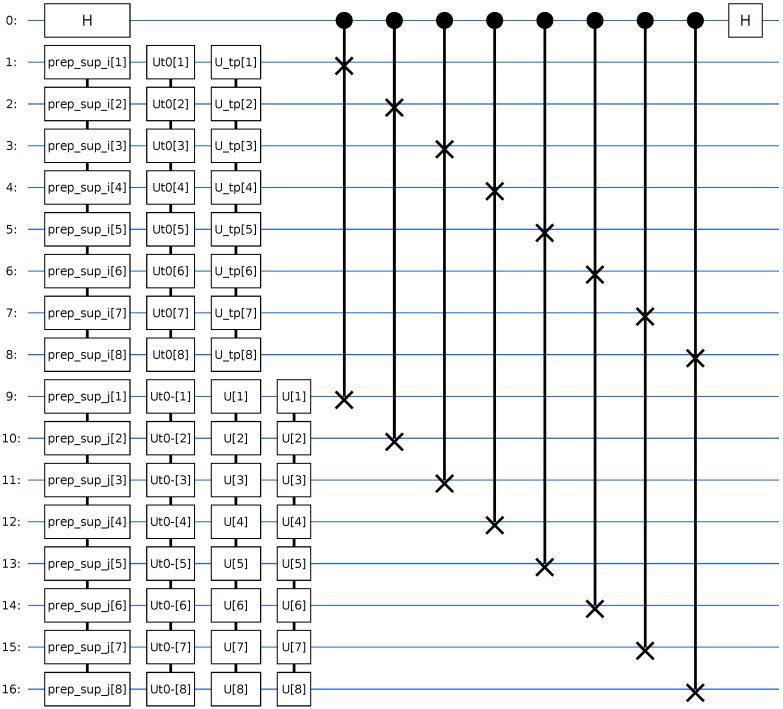
Multi-fidelity estimation (MFE) circuit used to extract the overlap amplitudes entering $C_{v_{p},\;v_{r}}\left(t\right)$ via SWAP tests on two $n_{\mathrm{sys}}$-qubit registers. One register is prepared in the superposition state $\left(|R\rangle +|\phi_{i}\rangle \right)/\sqrt{2}$ and the other in $\left(|R\rangle +|\phi_{j}\rangle \right)/\sqrt{2}$, where |R〉 is a fixed reference basis state and $|\phi_{i}\rangle$, $|\phi_{j}\rangle$ are the product and reactant wavepackets. Both registers undergo the same short-time Møller operator blocks and full time evolution, after which an ancilla-controlled SWAP test between the two registers yields the fidelities $F_{1}$ and $F_{2}$ used in the MFE reconstruction of the complex correlation amplitude $C_{v_{p},\;v_{r}}\left(t\right)$.

Both the modified Hadamard test and the multi-fidelity estimation (MFE) protocol reproduce the same time-dependent correlation structure within numerical precision. In particular, the short-time scattering peak, the long-time oscillatory behavior, and the relative phase evolution of the correlation function are consistently captured by both estimators. The MFE protocol achieves this without the use of controlled time evolution gates, as shown in Figure 8, significantly reducing the circuit depth while maintaining full complex overlap reconstruction; this reduction reflects an algorithmic advantage in scaling behavior rather than immediate suitability for NISQ hardware. This establishes MFE as a near-term-compatible alternative to the Hadamard test for computing scattering-type time correlation functions on digital quantum hardware.

### 5.6. Numerical Implementation

The circuit was implemented in Cirq [32] using dense matrix gates constructed from the kinetic plus London potential Hamiltonian on an 8 × 8(X, Y) bond coordinate grid. For each time step *t* in the range −1000 ≤ t ≤ 4000 a.u., the unitary propagator $U_{H}\left(t\right)\;=\;\exp\left(-i\hat{H}t\right)$ was computed using scipy.linalg.expm and wrapped as a controlled gate.

In the simulations reported here, we used a uniform bond coordinate grid with $N_{X}\;=\;N_{Y}\;=\;16$ points along each coordinate, spanning the interval $0\leq X,Y\leq X_{\max}\;=\;Y_{\max}\;=\;5\;a.u$. The resulting Hilbert-space dimension is $D=N_{X}N_{Y}=2^{n_{\mathrm{sys}}},$ so that the grid can be encoded exactly in an $n_{\mathrm{sys}}$-qubit register. The channel-resolved correlation functions $C_{\nu_{p},\nu_{r}}\left(t\right)$ were evaluated on a uniform time grid t∈[−1000, 4000]a.u., Δt=50a.u., which was sufficient to resolve both the short-time scattering peak near the barrier region and the long-time oscillatory recurrences in the asymptotic product channel.

The reactant and product scattering wavepackets $|\Psi_{r}\rangle$ and $|\Psi_{p}\rangle$ are loaded using the Cirq state preparation scheme. For each target state ψ on the *D*-dimensional grid, we construct a unitary $U_{\psi }$ such that $U_{\psi }|0\ldots 0\rangle =\psi$. The resulting D×D matrices $U_{r}$ and $U_{p}$ are then wrapped as MatrixGates in Cirq and reused at all times *t* inside the Hadamard and MFE circuits. This dense approach is not scalable to very large *D*, but it is sufficient for the modest grid sizes considered here and allows us to isolate the algorithmic features of the correlation estimation protocols.

The $D=N_{X}N_{Y}$ grid states are encoded in a register of $n_{\mathrm{sys}}=\log_{2}D$ qubits, such that each computational basis state corresponds to a single grid point (X, Y). For the grid size used here, this gives $n_{\mathrm{sys}}=8$ and D=256. The modified Hadamard test therefore acts on a total of $n_{\mathrm{tot}}=n_{\mathrm{sys}}+1=9$ qubits (one ancilla plus the $n_{\mathrm{sys}}$-qubit system register), whereas the MFE protocol uses two system registers and no ancilla, for a total of $n_{\mathrm{tot}}=2n_{\mathrm{sys}}=16$ qubits. These sizes are modest enough that we can represent the relevant state preparation and time evolution blocks as dense unitaries in the present proof-of-principle study, while still illustrating the scaling behavior of each algorithm.

**Figure 9:**
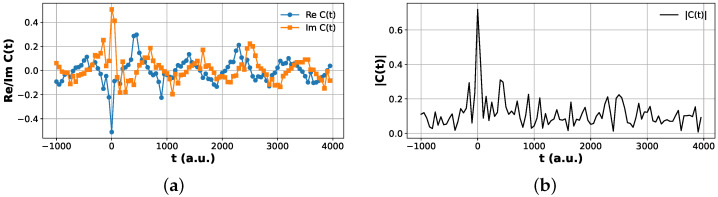
Correlation function obtained from the multi-fidelity estimation (MFE) protocol for the ground-state product channel. (**a**) Real and imaginary parts ReCvp=0,vr=0(t) and ImCvp=0,vr=0(t) as functions of time. (**b**) Magnitude $|C_{v_{p}=0,\;v_{r}=0}\left(t\right)|$ of the same correlation function, used in the subsequent energy-domain analysis. Excellent agreement with the Hadamard test and standard quantum calculations is observed throughout the propagation window.

**Figure 10:**
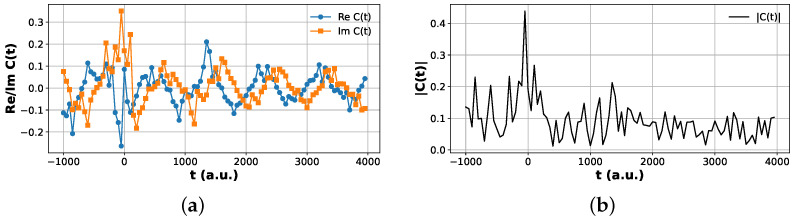
Correlation function obtained from the multi-fidelity estimation (MFE) protocol for the excited product vibrational channel $v_{p}=1$. (**a**) Real and imaginary parts ReCvp=1,vr=0(t) and ImCvp=1,vr=0(t) as functions of time. (**b**) Magnitude $|C_{v_{p}=1,\;v_{r}=0}\left(t\right)|$ of the same correlation function, used in the subsequent energy-domain analysis.

**Figure 11:**
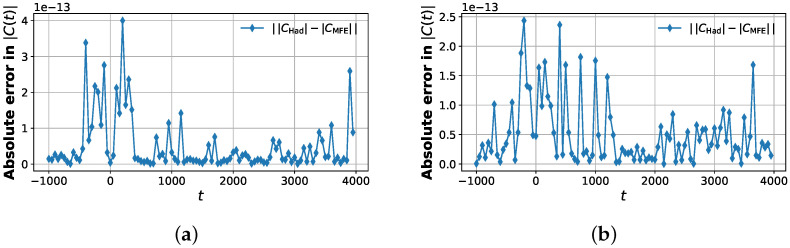
Absolute deviation between Hadamard and MFE correlation estimates. Time -dependent absolute error $|C_{\mathrm{Had}}\left(t\right)-C_{\mathrm{MFE}}\left(t\right)|$ for the channel-resolved correlation function over the full propagation window. (**a**) The left panel corresponds to the elastic channel $\left(\nu_{p},\;\nu_{r}\right)=\left(0,\;0\right)$ and (**b**) the right panel to the inelastic channel $\left(\nu_{p},\;\nu_{r}\right)=\left(1,\;0\right)$. The error remains on the order of $10^{-13}$ across all times, indicating machine precision agreement between the modified Hadamard test and the MFE protocol. The slightly larger fluctuations near t≈0 coincide with the transient scattering overlap where the correlation magnitude is maximal.

**Figure 12:**
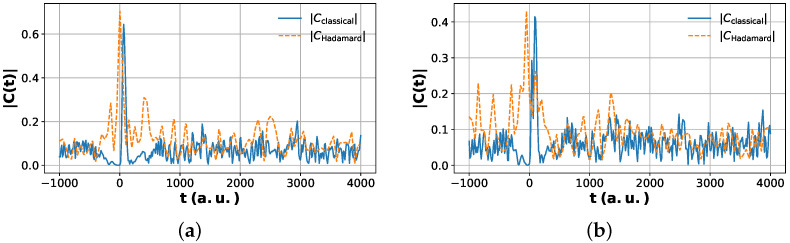
Comparison of standard quantum calculations and Hadamard test correlation magnitudes. Time-dependent magnitude of the correlation function |C(t)| obtained from standard quantum calculations for wavepacket propagation (solid blue) and from the modified Hadamard test quantum simulation (dashed orange). (**a**) The left panel corresponds to the transition into the product vibrational ground state $\nu_{p}^{\prime }=0$, while the (**b**) right panel shows the transition into the first excited product vibrational state $\nu_{p}^{\prime }=1$. In both channels, the Hadamard test results closely reproduce the short-time scattering peak near t≈0 and the subsequent long-time oscillatory behavior observed in the standard quantum calculation reference data.

All results shown in Figure 9, Figure 10, Figure 11 and Figure 12 were obtained using classical simulation of the quantum circuits and were not executed on physical quantum hardware. The purpose of these simulations is to validate the algorithmic construction and estimator equivalence in a controlled, noiseless setting. Hardware noise effects are therefore not included in the reported figures. Incorporating device-specific noise models and error mitigation strategies is an important direction for future work but is beyond the scope of this proof-of-principle algorithmic study.

## 6. Results Comparison

### 6.1. Hadamard vs. MFE

Figure 11 shows that the agreement between the modified Hadamard test and the MFE protocol is not only qualitative but quantitative at the level of numerical precision: the pointwise deviation $|C_{\mathrm{Had}}\left(t\right)-C_{\mathrm{MFE}}\left(t\right)|$ stays at the level of $10^{-13}$ over the entire propagation window. To quantify the global discrepancy, we computed discrete $\ell_{2}$ and $\ell_{\infty }$ norms of the difference $|C_{\mathrm{Had}}\left(t\right)|-|C_{\mathrm{MFE}}\left(t\right)|$ over all time samples. For the elastic channel $\left(\nu_{p},\;\nu_{r}\right)=\left(0,\;0\right)$, we obtain

$${∥\;|C_{\mathrm{Had}}\left|-\right|C_{\mathrm{MFE}}|\;∥}_{2}\approx 6.38\times 10^{-12},\;{∥\;|C_{\mathrm{Had}}\left|-\right|C_{\mathrm{MFE}}|\;∥}_{\infty }\approx 4.00\times 10^{-13},$$

while, for the inelastic channel $\left(\nu_{p},\;\nu_{r}\right)=\left(1,\;0\right)$, the corresponding values are

$${∥\;|C_{\mathrm{Had}}\left|-\right|C_{\mathrm{MFE}}|\;∥}_{2}\approx 5.25\times 10^{-12},\;{∥\;|C_{\mathrm{Had}}\left|-\right|C_{\mathrm{MFE}}|\;∥}_{\infty }\approx 2.44\times 10^{-13}.$$

These norms are at or below double-precision roundoff and many orders of magnitude smaller than the typical correlation magnitude ($|C_{\nu_{p},\nu_{r}}\left(t\right)|∼10^{-1}$ near the short-time peak). Thus, the ancilla-free MFE construction reproduces the overlap estimated by the deeper, ancilla-controlled Hadamard test to machine precision in both scattering channels. Since MFE eliminates the need for a global controlled U(t) and replaces it with SWAP tests and simpler time evolutions, this matching accuracy at dramatically reduced circuit overhead is a key algorithmic advantage, consistent with depth reduction arguments in the literature.

### 6.2. Standard Quantum vs. Quantum Circuit Correlations

As a basic consistency check, we verify that the quantum circuit-evaluated correlation at t=0, $C\left(0\right)=\langle \Psi^{-}|\Psi^{+}\rangle ,$ agrees with the direct inner product $\langle \Psi_{p}|\Psi_{r}\rangle$ of the prepared reactant and product wavepackets on a classical computer. For both channels, the real and imaginary parts of C(0) agree with the standard quantum mechanical reference to better than $10^{-12}$, confirming that the state preparation and overlap estimation procedures are implemented correctly.

Figure 11 compares the modified Hadamard test and the multi-fidelity estimation (MFE) protocol for the present problem. The two estimators show excellent numerical agreement, with residual differences at the level of floating-point roundoff, indicating that the ancilla-free MFE protocol reproduces the same correlation values as the Hadamard test within the precision accessible here. Figure 12 places this algorithmic agreement in a physical context by comparing the quantum circuit-computed correlation magnitudes with the standard quantum calculation wavepacket reference.

The pronounced peak in $|C_{\nu_{p},\nu_{r}}\left(t\right)|$ near t≈0 arises when the reactant- and product-like wavepackets overlap in the interaction region, providing a direct time-domain signature of the scattering event. At later times, the correlation magnitude exhibits weaker, quasi-periodic oscillations whose structure depends on the product channel. These long-time features originate from vibrational recurrences of the bound H-H pair in the product well and are consistent with established standard quantum mechanical calculations on classical computer wavepacket descriptions of the H + H_2_ exchange reaction. Small quantitative deviations between the quantum circuit and standard quantum calculations curves can be attributed to the finite grid resolution and statistical sampling effects.

Taken together, these results show that (i) the MFE protocol reproduces the complex time-dependent correlation function with accuracy comparable to the Hadamard test for the present simulations and (ii) the quantum-computed correlations capture the essential dynamical features of the scattering process—both the transient reaction peak and the channel-dependent long-time oscillations—while remaining quantitatively close to high-resolution standard quantum calculations on classical computer wavepacket results.

To validate the correctness of the quantum circuit implementation independently of any specific software backend, we performed several internal consistency checks. First, the exact identity $C\left(0\right)=\langle \Psi^{-}|\Psi^{+}\rangle$ was verified by comparing the circuit-evaluated overlap at t=0 with the direct inner product of the prepared wavepackets, with agreement better than $10^{-12}$. Second, the modified Hadamard test and the multi-fidelity estimation (MFE) protocol—two algorithmically distinct estimators—were shown to reproduce near-identical correlation functions. These checks relied on exact algebraic relations and estimator equivalence and therefore provide validation beyond agreement with external standard quantum calculations on a classical computer propagation code.

## 7. Algorithmic Complexity and Error Sources

In the present work, time evolution under the full Hamiltonian *H* and the asymptotic Hamiltonian $H_{0}$ is implemented by explicitly constructing dense unitary propagators. The time evolution operator $U\left(t\right)=e^{-iHt}$ is evaluated numerically via matrix exponentiation and then wrapped as a MatrixGate in the Cirq framework. This avoids Trotter–Suzuki or product formula decompositions at the circuit level but shifts the main computational bottleneck to the cost of generating and storing dense matrices on a classical processor.

This choice was made for benchmarking purposes: since the Hilbert space remains classically tractable, we can compute U(t) exactly using matrix exponentiation and validate the quantum circuit output against high-precision classical simulations of standard quantum calculations. This approach ensures that all observed differences arise from quantum circuit behavior and not from numerical artifacts in the classical simulations of standard quantum calculations.

Looking ahead, this method is expected to provide significant advantages in regimes where direct classical simulations of standard quantum calculations become infeasible. As the system size increases, the cost of storing and applying U(t) as a dense matrix scales exponentially, while a quantum computer can implement the same operator through sequences of native gates (e.g., via Trotterization or qubitization) with polynomial overhead. In such cases, our correlation function-based algorithm can be used to simulate correlation dynamics intractable for classical simulations of standard quantum calculations, offering a clear path toward a quantum advantage.

We emphasize that these numerical experiments are intended as conceptual demonstrations of the proposed algorithms on small-scale instances using classical simulators of quantum circuits, rather than as full software performance benchmarks.

### 7.1. Challenges of Controlled Unitaries in Hadamard Test

The Hadamard test requires controlled versions of both the propagation operator U(t) and the state preparation unitaries $U_{\gamma }$ and $U_{\gamma^{\prime }}$ that map the vacuum state to reactant and product wavepackets. Mathematically,

(37) $$\langle \Psi_{\gamma^{\prime }}^{-}\left|U\left(t\right)\right|\Psi_{\gamma }^{+}\rangle =\langle 0|U_{\gamma^{\prime }}^{†}\;U\left(t\right)\;U_{\gamma }|0\rangle ,$$

so, in circuit form, each *U* must be promoted to a controlled operation [33]. If a dense matrix gate acts on *n* system qubits, its controlled version requires n+1 qubits and a substantially larger number of two-qubit entangling gates. As *D* grows, this leads to a sharp increase in both the gate count and circuit depth, even when U(t) has been precomputed classically [34].

#### Sampling Error in the Hadamard Test

In the Hadamard test implementation, errors arise from statistical sampling during the execution of the quantum circuit. At each time point *t* in the correlation grid, the estimator $\hat{C}\left(t\right)$ obtained from *N* circuit repetitions (shots) fluctuates around the exact value C(t) due to measurement noise. This sampling error is bounded by a Chernoff bound so that the number of samples required to estimate the correlation function $C_{\gamma^{\prime },\gamma }\left(t\right)$ with additive precision ϵ and a success probability of at least 1−δ scales as $N=O\;\left(\frac{\ln(1/\delta )}{\epsilon^{2}}\right).$ Thus, the measurement overhead grows polynomially in 1/ϵ but is independent of the Hilbert-space dimension for a fixed circuit depth.

The rapid growth in circuit depth associated with controlled unitaries is the primary motivation for the multi-fidelity estimation (MFE) protocol. By eliminating global controlled time evolution, MFE provides a pathway for scaling correlation function estimation even when controlled versions of large unitaries become prohibitively expensive.

### 7.2. Matrix Exponentiation and Storage Cost

For a bond coordinate grid of dimension $D=N_{X}N_{Y}$, the Hamiltonian is represented as a D×D sparse matrix. Computing the exact propagator $U\left(t\right)=e^{-iHt}$ by dense matrix exponentiation requires $O(D^{3})$ operations using standard classical linear algebra routines, together with $O(D^{2})$ memory to store the resulting dense matrix. This cost is manageable for the modest grid sizes (D≲64) employed in our demonstrations but becomes prohibitive for larger discretizations. In practice, the present implementation is therefore limited primarily by classical computer precomputation rather than by the quantum circuit depth.

### 7.3. Circuit Depth Estimate for MFE

We briefly quantify the reduction in circuit depth afforded by the multi-fidelity estimation (MFE) protocol relative to the Hadamard test. Consider a unitary time evolution operator $U=e^{-i\hat{H}t}$ acting on *N* qubits, decomposed into a circuit containing *p* CNOT gates and *q* single-qubit gates. In the Hadamard test, implementing the corresponding controlled unitary requires *p* Toffoli gates and *q* controlled single-qubit gates. After decomposing each Toffoli gate into CNOTs, the controlled unitary contains between 3p and 6p CNOTs, in addition to further two-qubit and single-qubit operations.

By contrast, the MFE protocol requires only (p+r) CNOTs, where *r* denotes the number of CNOT gates needed to prepare the reference superposition state. Consequently, although the multiplicative factor in front of *p* in the Hadamard test may appear modest, it substantially limits the achievable circuit depth. Under comparable assumptions, the MFE protocol can therefore enable between three and six times more time steps than the Hadamard test on the same hardware.

Table 1 summarizes the circuit resources required by the two estimators and provides a direct, engineering-level comparison beyond asymptotic depth arguments.

## 8. Discussion and Conclusions

In summary, this work demonstrates that digital quantum circuits can reproduce channel-resolved correlation overlaps for the benchmark H + H_2_ exchange reaction. Both an ancilla-based Hadamard test and the ancilla-free multi-fidelity estimation (MFE) protocol yield numerically indistinguishable correlation functions. The resulting time-dependent overlaps capture the same transient peak and long-time vibrational recurrences observed in classical simulations of standard quantum calculations of wavepackets on an LEPS potential energy surface.

From an algorithmic standpoint, our results show that time-dependent Møller correlation functions can be evaluated on a gate-based quantum computer using two complementary strategies. The modified Hadamard test directly encodes the complex overlap into the expectation value of a single ancilla qubit but requires controlled implementations of the full time evolution operator $U\left(t\right)=e^{-iHt}$ and of the state preparation unitaries. The MFE protocol removes these global controlled operations in favor of a sequence of SWAP tests and separate evolutions of differently prepared registers. The error analysis (Figure 11) demonstrates that the resulting correlation functions agree to machine precision, establishing MFE as a depth-reduced, ancilla-free alternative to the Hadamard test that is particularly attractive for near-term hardware with limited coherence times and noisy two-qubit gates.

From a chemical physics standpoint, the time traces of $C_{\nu_{p},\nu_{r}}\left(t\right)$ provide a compact dynamical fingerprint of the reaction [2,5,6]. The pronounced peak near t≈0 in both channels corresponds to the regime where the incoming reactant and outgoing product wavepackets overlap in the interaction region, i.e., the scattering event itself. At later times, the correlation magnitude settles into weaker, quasi-periodic oscillations whose structure depends on the product channel. For the elastic channel $\nu_{p}=0$, these oscillations are relatively strong and regular, consistent with coherent vibrational motion of the bound H-H product [35]. For the inelastic channel $\nu_{p}=1$, the overall amplitude is reduced and the pattern more irregular, reflecting the smaller Franck–Condon overlap between the initial reactant packet and the first excited vibrational state of the product. Figure 12 shows that the quantum results reproduce all of these features, indicating that the quantum circuits not only match the classical simulation of standard quantum calculation benchmark numerically but also faithfully encode the underlying reaction dynamics.

More broadly, the present results position time-domain Møller correlation functions as a practical and chemically meaningful intermediate target for the quantum simulation of molecular dynamics. Rather than attempting full state tomography or the direct solution of the Schrödinger equation on a large grid, the algorithm focuses on physically relevant overlaps that can be accessed through relatively shallow circuits. Extending the present approach to higher-dimensional grids, more accurate three-dimensional H_3_ potential energy surfaces, and larger reactive systems where classical simulations of standard quantum calculations of wavepacket propagation become prohibitive is a natural next step [1,17].

## Figures and Tables

**Table 1:** Circuit resource comparison between the modified Hadamard test and the multi-fidelity estimation (MFE) protocol for the instances considered in this work. Gate counts are reported after decomposition into single-qubit rotations and CNOTs.

	Hadamard Test	MFE
System qubits	$n_{\mathrm{sys}}$	$2n_{\mathrm{sys}}$
Ancilla qubits	1	0
Controlled-U(t) blocks	Yes	No
Controlled state preparation	Yes	No
Estimated CNOT count	∼O(p)	∼O(p+r)
Circuit depth (qualitative)	High	Reduced

## Data Availability

The code required to reproduce the numerical results and figures in this manuscript is openly available in the GitHub repository: https://github.com/IshmamShah/Correlations (accessed on 26 January 2026).
